# The Interference of Selected Cytotoxic Alkaloids with the Cytoskeleton: An Insight into Their Modes of Action

**DOI:** 10.3390/molecules21070906

**Published:** 2016-07-12

**Authors:** Xiaojuan Wang, Mine Tanaka, Sonja Krstin, Herbenya Silva Peixoto, Michael Wink

**Affiliations:** Department of Biology, Institute of Pharmacy and Molecular Biotechnology, Heidelberg University, INF 364, D-69120 Heidelberg, Germany; mine_tanaka@hotmail.com (M.T.); Krstin@uni-heidelberg.de (S.K.); hspeixoto1@gmail.com (H.S.P.); wink@uni-heidelberg.de (M.W.)

**Keywords:** alkaloids, sanguinarine, chelerythrine, chelidonine, noscapine, protopine, homoharringtonine, paclitaxel, microtubules, actin filaments

## Abstract

Alkaloids, the largest group among the nitrogen-containing secondary metabolites of plants, usually interact with several molecular targets. In this study, we provide evidence that six cytotoxic alkaloids (sanguinarine, chelerythrine, chelidonine, noscapine, protopine, homoharringtonine), which are known to affect neuroreceptors, protein biosynthesis and nucleic acids, also interact with the cellular cytoskeleton, such as microtubules and actin filaments, as well. Sanguinarine, chelerythrine and chelidonine depolymerized the microtubule network in living cancer cells (Hela cells and human osteosarcoma U2OS cells) and inhibited tubulin polymerization in vitro with IC_50_ values of 48.41 ± 3.73, 206.39 ± 4.20 and 34.51 ± 9.47 μM, respectively. However, sanguinarine and chelerythrine did not arrest the cell cycle while 2.5 μM chelidonine arrested the cell cycle in the G_2_/M phase with 88.27% ± 0.99% of the cells in this phase. Noscapine and protopine apparently affected microtubule structures in living cells without affecting tubulin polymerization in vitro, which led to cell cycle arrest in the G2/M phase, promoting this cell population to 73.42% ± 8.31% and 54.35% ± 11.26% at a concentration of 80 μM and 250.9 μM, respectively. Homoharringtonine did not show any effects on microtubules and cell cycle, while the known microtubule-stabilizing agent paclitaxel was found to inhibit tubulin polymerization in the presence of MAPs in vitro with an IC_50_ value of 38.19 ± 3.33 μM. Concerning actin filaments, sanguinarine, chelerythrine and chelidonine exhibited a certain effect on the cellular actin filament network by reducing the mass of actin filaments. The interactions of these cytotoxic alkaloids with microtubules and actin filaments present new insights into their molecular modes of action.

## 1. Introduction

Alkaloids, the largest class of nitrogen-containing secondary metabolites that occur mainly in plants, animals, bacteria and fungi, have a great diversity of structures and exhibit a wide range of pharmacological properties, such as antibacterial, antiviral, anti-inflammatory and antitumor effects [1,2,3,4,5]. The biological activities of alkaloids are mainly due to their interactions with various molecular targets, such as biomembranes, proteins, DNA topoisomerase, nucleic acids, neuroreceptors and ion channels, among which the cytoskeleton represents one of the most important targets [1,6].

Microtubules and actin filaments, the two essential elements of the cytoskeleton in eukaryotic cells, have multiple functions in cellular processes: microtubules decide the spatial distribution of membrane-enclosed organelles and form mitotic spindles during the cell cycle, while actin filaments determine the cell shape and together with microtubules are responsible for cell motility [7,8]. The importance of microtubules and actin filaments in biological activities, such as vesicular and organelle transport, cell and nuclear migration, cell proliferation and division, makes them attractive targets for natural toxins in cancer research [9,10,11].

Microtubule-binding agents (MBAs) disrupt the structure and dynamics of microtubules, which arrest the cell cycle and eventually lead to cell apoptosis [12]. MBAs are important components in combination chemotherapy and widely used in clinics to treat many kinds of cancers, such as leukemia, lymphomas and solid tumors [13,14]. Alkaloids constitute the most important group of MBAs; well-known examples are the diterpene alkaloids paclitaxel and docetaxel (toxins from *Taxus*), which stabilize the polymerized microtubule, and the monoterpene indole alkaloids vinblastine, vincristine and vinorelbine (vinca alkaloids from *Catharanthus roseus*), which depolymerize microtubules or prevent tubulin assembly [15,16].

The structure and dynamics of actin filaments can be stabilized by the hexapeptide phalloidin (a toxic alkaloid from *Amanita phalloides*) or destabilized by the alkaloids cytochalasin (fungal toxins) and latrunculin (toxins from marine sponges) [1,6,17]. Actin filaments, like microtubules, should be a good target for toxins. However, only a few drugs have been developed to target the actin system [1].

Due to their intricate ring systems carrying active groups, which have been modified during evolution, alkaloids usually interact with several molecular targets [1]. Our previous study [18] has provided evidence that the tryptophan-derived quinoline alkaloids camptothecin and topotecan, which are known DNA topoisomerase I inhibitors, additionally interact with cytoskeleton. Alkaloids with multitarget properties show great potential in the future development of anticancer therapies. However, their corresponding mechanisms are not yet fully or correctly understood.

**Figure 1:**
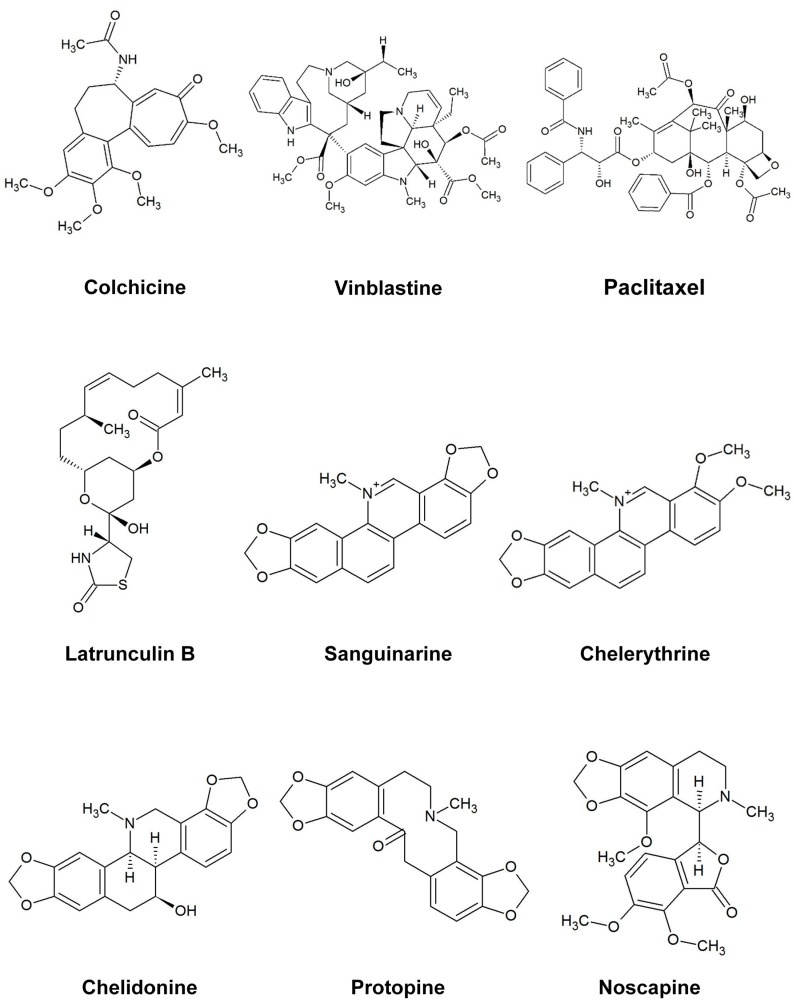
The structure of the alkaloids and reference drugs tested in the study.

In this study, six cytotoxic alkaloids (Figure 1) affecting different targets were tested to explore their effects on microtubules and actin filaments in vivo (Hela: human cervical cancer, MCF-7: human breast cancer and U2OS: human osteosarcoma cancer cells) by use of GFP markers and immunostaining. Furthermore, we analyzed their interaction with tubulin in vitro employing isolated tubulin in a polymerization assay. Modes of action include DNA intercalation and suppression of NF-κB activation (sanguinarine, a benzophenanthridine alkaloid from *Sanguinaria canadensis*) [19], DNA intercalation and inhibition of protein kinase c (chelerythrine, a benzophenanthridine alkaloid from *Chelidonium majus*) [20], inhibition of telomerase and tubulin (chelidonine, a benzophenanthridine alkaloid from *Chelidonium* *majus*) [6], agonist of σ site and antagonist of BK receptor (noscapine, a phthalide isoquinoline alkaloid from *Papaver somniferum*) [21,22], reduction of intracellular calcium flux (protopine, a benzylisoquinoline alkaloid from *Chelidonium majus*) [23] and inhibition of protein synthesis (homoharringtonine, a cephalotaxine alkaloid from *Cephalotaxus harringtonia*) [24]. Other microtubule-binding agents, such as colchicine, vinblastine, paclitaxel and the actin-binding agent latrunculin B, were studied as positive controls for comparison. In this study we can provide evidence for partly unknown interactions between these alkaloids and the cytoskeleton.

## 2. Results

### 2.1. Anti-Proliferative Activity of Alkaloids

To study the effects of selected alkaloids on the proliferation of human cancer cell lines, we investigated their cytotoxicity in Hela, MCF-7 and U2OS cells (Table 1). Known microtubule-binding agents, such as colchicine, vinblastine and paclitaxel, showed significant anti-proliferative effects against all three cell lines. Whereas vinblastine exhibited the strongest inhibition with IC_50_ values between 0.02 and 0.10 nM, homoharringtonine caused the second strongest cytotoxicity with IC_50_ values between 0.87 and 9.03 nM. The benzophenanthridine alkaloids sanguinarine, chelerythrine and chelidonine are also cytotoxic; they inhibited the growth of three cell lines with IC_50_ values ranging between 0.92 and 7.63 μM. Noscapine and protopine exhibited a 10–20-times lower cytotoxicity than the other alkaloids. The known actin filament binding latrunculin B showed IC_50_ values between 5.5 and 36.8 µM (Table 1).

### 2.2. Do Alkaloids Interfere with Microtubules in Living Cells?

#### 2.2.1. Influence on Microtubules 

**Figure 2:**
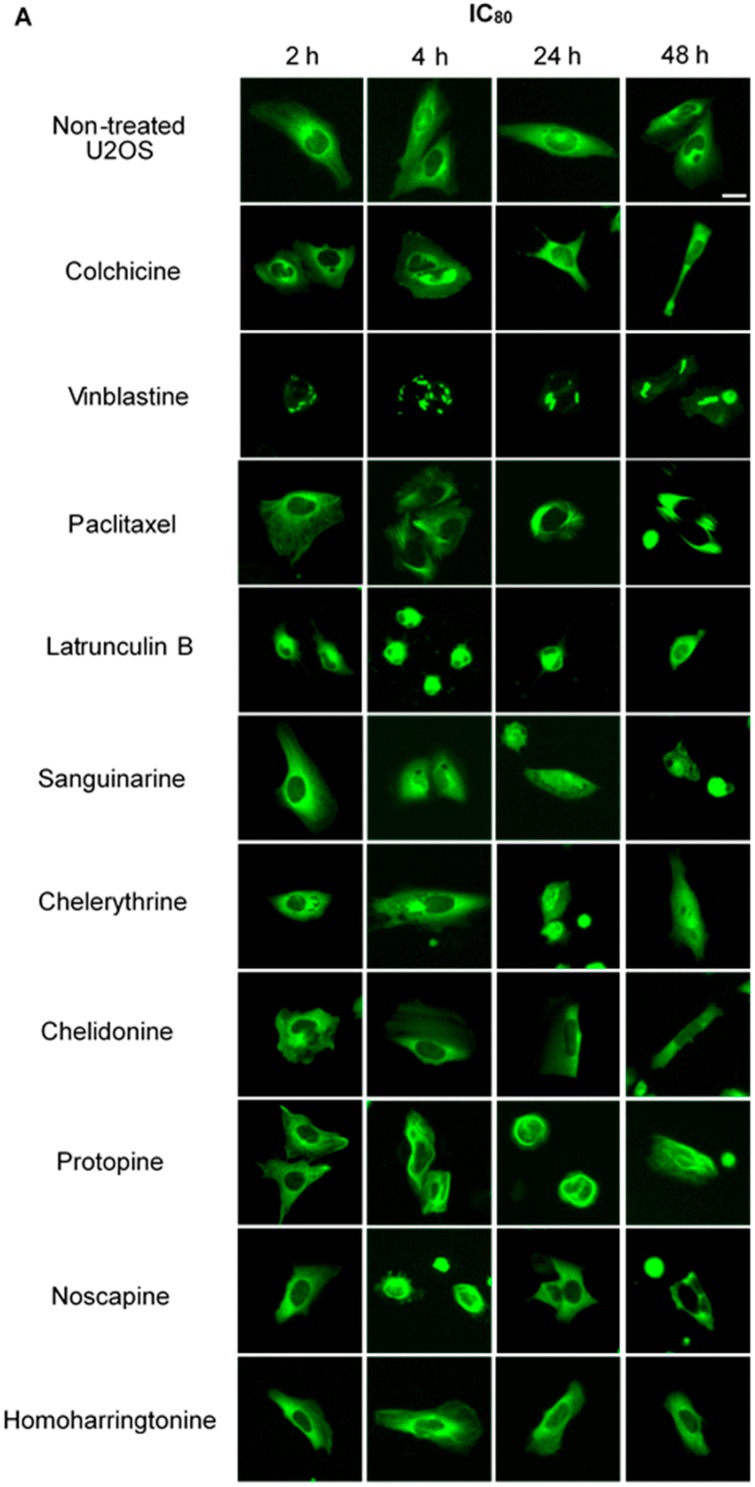

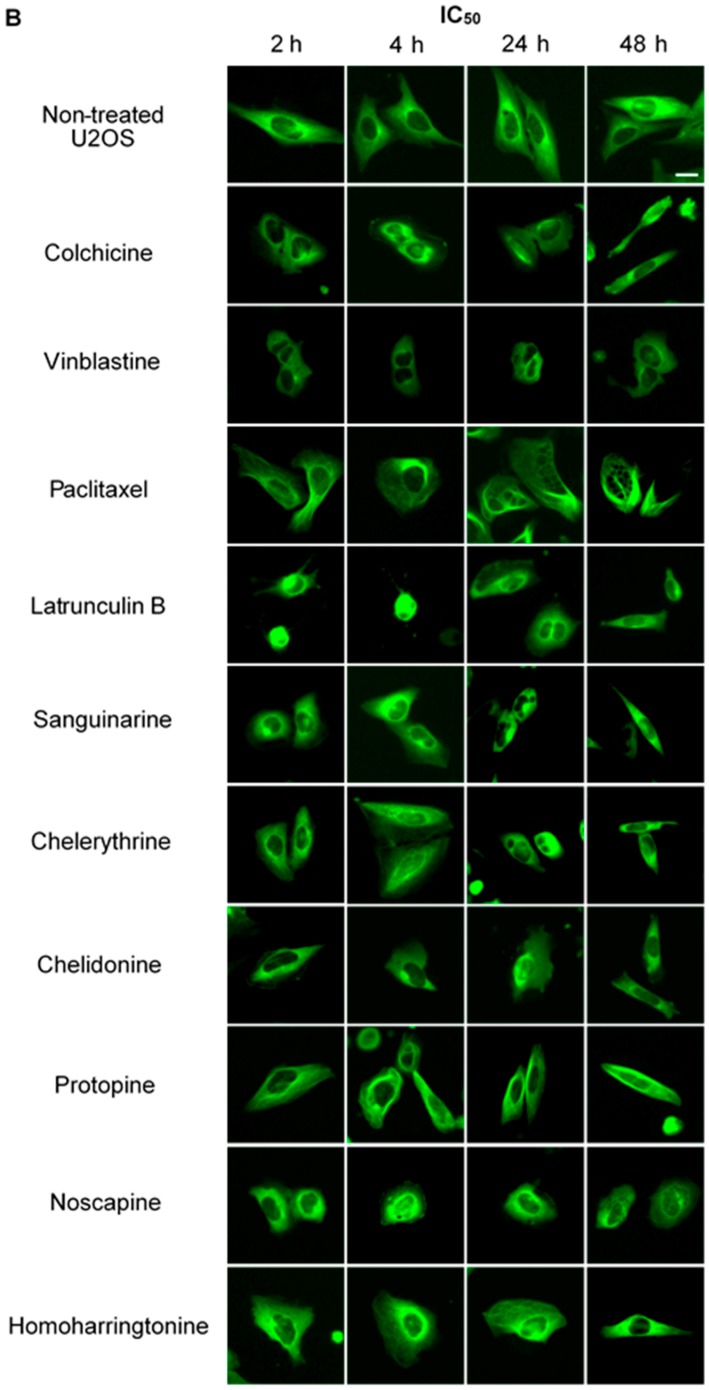
The effects of tested alkaloids on the microtubule network in U2OS cells expressing GFP-labeled tubulin. Panels show micrographs of U2OS cells treated for 2 h, 4 h, 24 h and 48 h with all ten alkaloids at concentrations of IC_80_ (**A**) and IC_50_ (**B**). The known tubulin inhibitors colchicine induced microtubule depolymerization, vinblastine caused tubulin paracrystals and paclitaxel enhanced microtubule depolymerization. Actin-binding agent latrunculin B caused the rapid change of cell morphology. Bar = 10 μm.

We treated U2OS cells, which contain α-tubulin-GFP, with different concentrations of the six alkaloids to determine their potential interaction with microtubules. Figure 2 illustrates the dose dependence of each alkaloid on the microtubule network. In control cells, the microtubules extended continuously through the cytoplasm and formed an extensive intracellular network with the exception of the nuclear region. Treatment with known MBAs induced microtubule depolymerization or aggregation: the tubulin-binding drug colchicine reduced the mass of the microtubule network, which was less dense at the cell periphery compared to non-treated cells. Vinblastine (also a tubulin- binding anticancer drug) differed from colchicine in that the microtubules were extensively depolymerized and tubulin paracrystals were formed and dispersed through the cytoplasm. The microtubule stabilizing drug paclitaxel promoted the polymerization of microtubules; their brightness and thickness increased with time. The actin-binding agent latrunculin B immediately changed the cell morphology, and the cells returned to the normal morphology after 24 h of incubation. The benzophenanthridine alkaloids significantly altered the microtubule network: sanguinarine disrupted microtubule distribution and altered their morphology, resulting in many spherical cavities in the microtubule network. Specifically, an abnormal aggregation of microtubules could be seen around the nucleus. Chelerythrine displayed similar effects as sanguinarine, with many globular cavities appearing in the network and aberrant microtubules assembling around the nucleus. Chelidonine significantly decreased cellular microtubule mass (similar to colchicine). Noscapine remarkably depolymerized the microtubule network and reduced its mass, while protopine notably enhanced the assembly of the microtubule network, altered its distribution and changed the cell morphology. No obvious changes on the cellular microtubule network were observed after the treatment of the cells with the protein biosynthesis inhibitor homoharringtonine (Figure 2).

#### 2.2.2. Influence on Spindle Apparatus 

**Figure 3:**
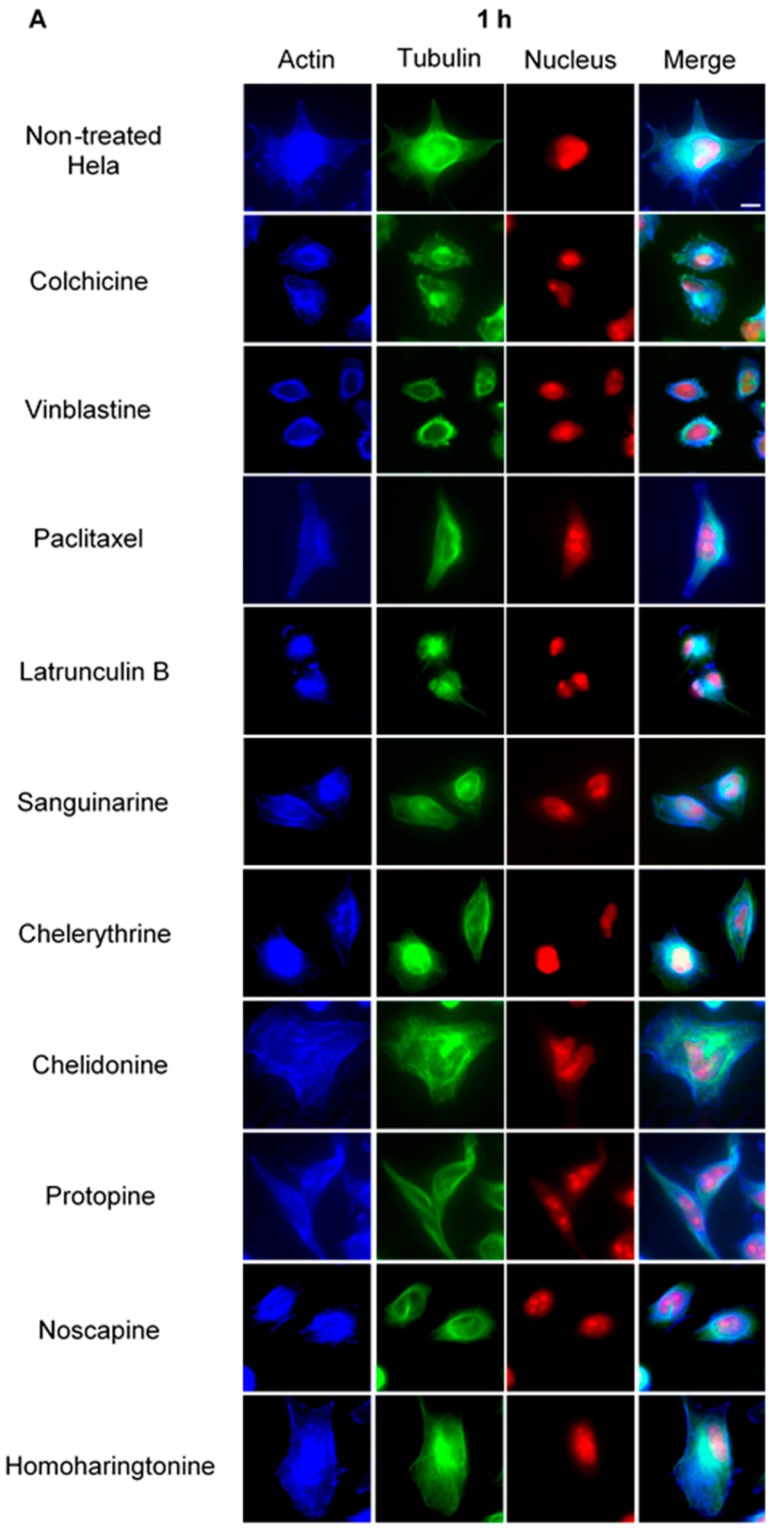

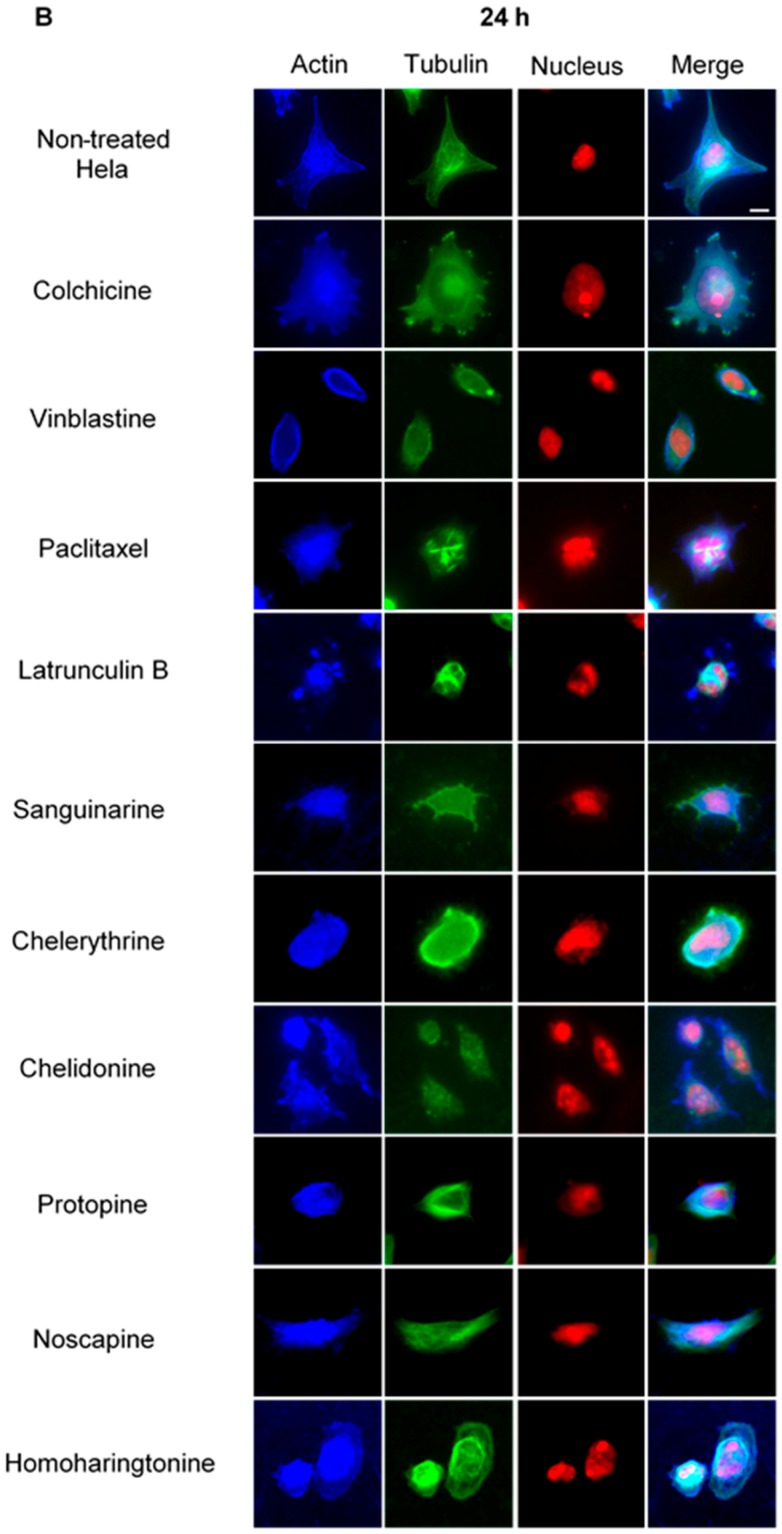
The effects of alkaloids on Hela cellular microtubules using immunofluorescence staining. Microtubules were stained by mouse anti-α-tubulin monoclonal IgG and goat anti-mouse IgM-FITC (green); actin filaments were stained by Atto 390 phalloidin (blue); and nucleus was stained by propidium iodide (red). Panels show immunofluorescence micrographs of Hela interphase cells treated for 1 h (**A**) and 24 h (**B**) with all ten compounds at the concentration of IC_80_. The known tubulin inhibitors colchicine induced microtubule depolymerization, vinblastine caused tubulin paracrystals and paclitaxel enhanced microtubule polymerization. Actin-binding agent latrunculin B caused the change of cell morphology and depolymerization of actin filaments. Bar = 10 μm.

**Figure 4:**
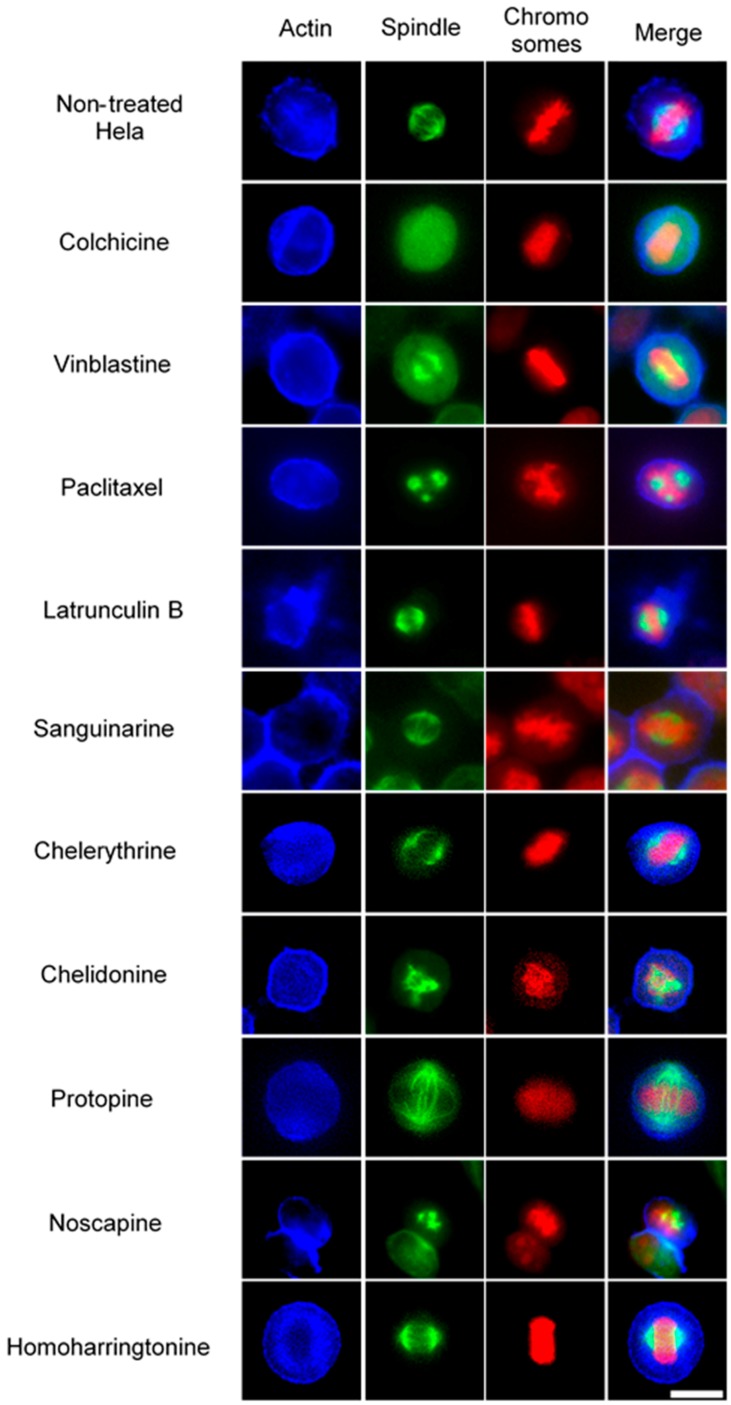
The effects of alkaloids on Hela metaphase cells using immunofluorescence staining. Panels show immunofluorescence micrographs of Hela interphase cells treated for 24 h with all ten compounds at the concentration of IC_50_. Absent spindles, depolymerized bipolar spindles and aberrant spindles were caused by colchicine, vinblastine and paclitaxel, respectively. Bar = 10 μm.

Using immunofluorescence staining, we further characterized the effects of the alkaloids on mitotic microtubules in Hela cells (Figure 3 and Figure 4). The effects of colchicine, paclitaxel, latrunculin B, chelidonine, noscapine, protopine and homoharringtonine on interphase cellular microtubules were comparable to the findings in U2OS cells, while tubulin paracrystal formation was lower in Hela cells than in U2OS cells after vinblastine treatment. Sanguinarine and chelerythrine, which produced many spherical cavities in microtubule network in U2OS cells, significantly reduced the mass of microtubules in Hela cells.

In non-treated metaphase Hela cells (Figure 4), most of the mitotic cells showed a bipolar spindle with all chromosomes well aligned in the metaphase plate and actin filaments dispersed throughout the cytoplasm. Compacted chromosomes with a completely depolymerized spindle appeared in colchicine-treated cells, while depolymerized bipolar spindles were observed in vinblastine-treated cells. Paclitaxel induced aberrant, but still bipolar spindles with chromosomes cluttering around them. Sanguinarine, which significantly affected the cellular microtubule network both in U2OS and Hela cells, had no impact on mitotic spindles. Chelerythrine caused abnormal, but still bipolar spindles, while chelidonine and noscapine both led to multipolar spindles with chromosome congression failure. Differing from the other alkaloids, protopine increased tubulin assembly, and thickened spindle arrays can be seen in Figure 4. Similar to sanguinarine, homoharringtonine did not affect mitotic spindles.

### 2.3. Benzophenanthridine Alkaloids Inhibited Tubulin Polymerization in Vitro

The previous results (Figure 2, Figure 3 and Figure 4) indicated a potential interaction between tested alkaloids and microtubules or tubulin. In order to understand the mechanisms, we examined the effects of these alkaloids on the polymerization of isolated tubulin subunits into microtubules *in vitro*. As shown in Table 2, sanguinarine, chelerythrine and chelidonine significantly inhibited the tubulin assembly with IC_50_ values of 48.41 ± 3.73, 206.39 ± 4.20 and 34.51 ± 9.47 μM, respectively. The known tubulin-binding drugs colchicine and vinblastine showed a more pronounced inhibition with IC_50_ values of 2.89 ± 0.36 μM and 1.42 ± 0.05 μM, respectively. Paclitaxel, the known microtubule-stabilizing agent, also prevented tubulin polymerization with an IC_50_ value of 38.19 ± 3.33 μM. Latrunculin B, noscapine, protopine and homoharringtonine did not affect tubulin polymerization.

**Figure 5:**
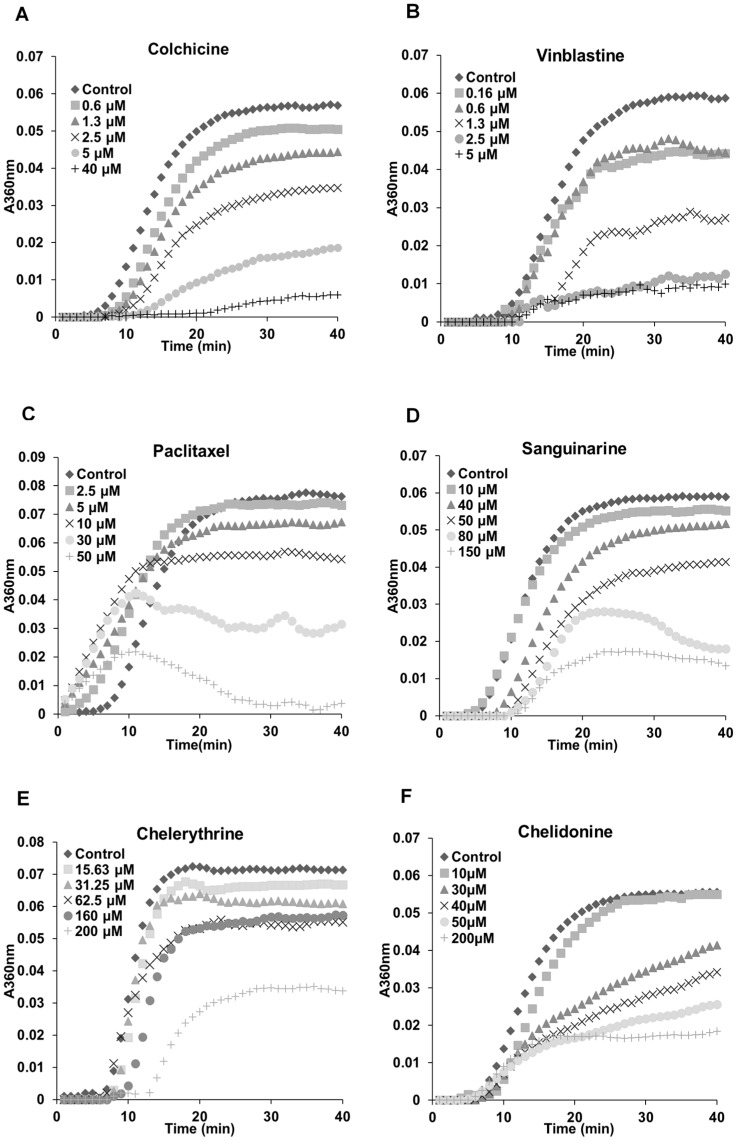
Benzophenanthridine alkaloids inhibited tubulin polymerization in vitro. Polymerization of tubulin with MAPs in the assembly buffer was measured in the absence (◆) and in the presence of different concentrations of compounds. (**A**,**B**,**D**–**F**) The inhibition of nucleation and growth phase during the microtubule assembly caused by colchicine, vinblastine, sanguinarine, chelerythrine and chelidonine. (**C**) A different mode of action of paclitaxel in which it accelerated the formation of nucleation and inhibited the growth phase during the assembly.

The polymerization dynamics of tubulin after treatment with each alkaloid are documented in Figure 5. In the absence of compounds, the polymerization begins with a slow nucleation start, which is then followed by the rapid growth of the microtubule polymer. When the addition of the new subunits balances the removal of the old subunits, the polymerization reaches the equilibrium phase, which is known as “tread milling” [25,26]. The effects of sanguinarine, chelerythrine and chelidonine on tubulin assembly were similar (although weaker) to colchicine and vinblastine; the time needed for nucleation was longer, and the growth phase was inhibited. A slower growth of the microtubule polymer was observed, and the system approached the steady state sooner. The mode of action of paclitaxel was different in that it accelerated the formation of nucleation and inhibited the growth phase during the assembly.

### 2.4. Influence of Benzophenanthridine Alkaloids on Actin Filaments in Hela Cells

**Figure 6:**
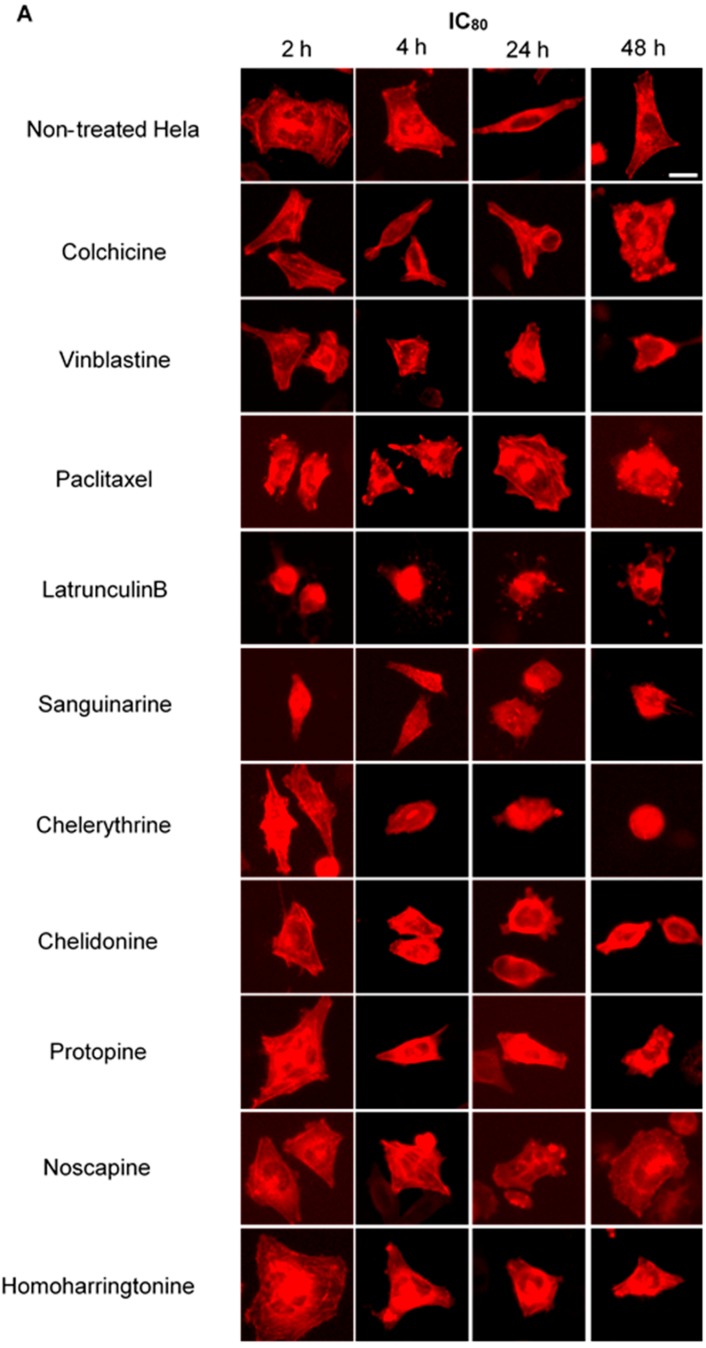

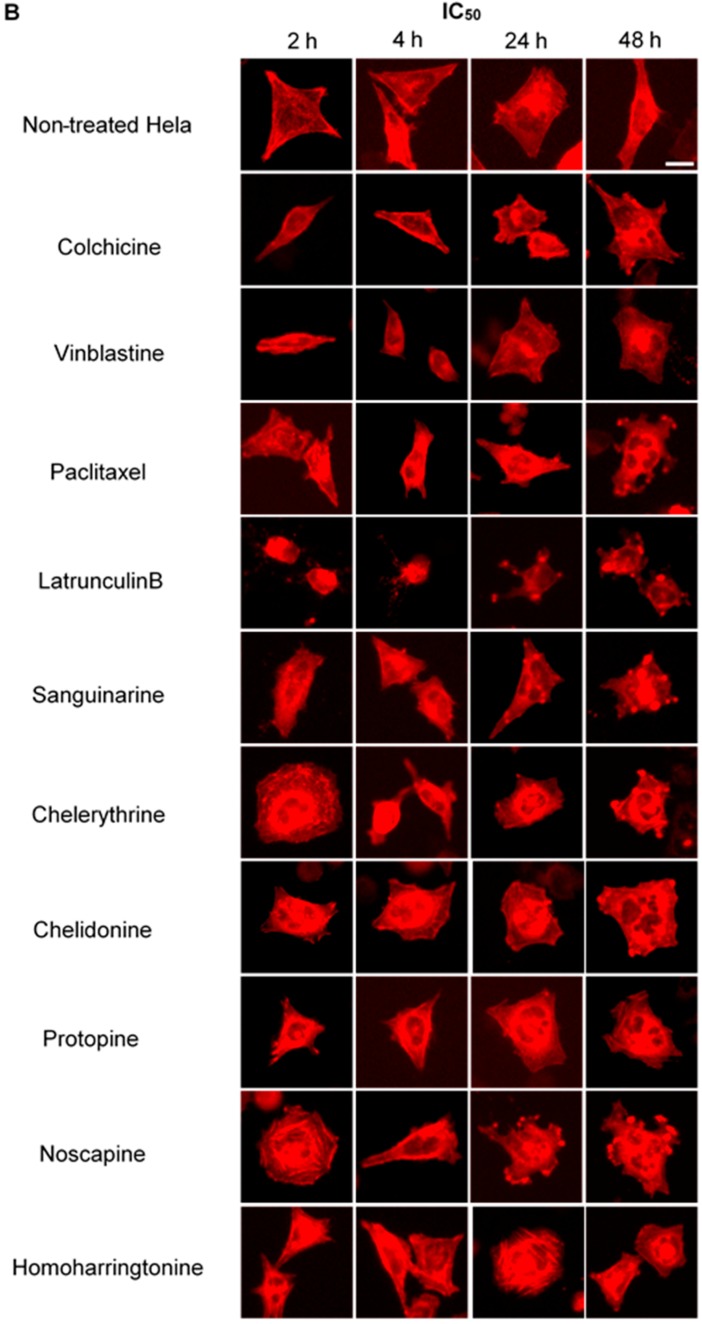
Benzophenanthridine alkaloids reduced the mass of actin filaments at a high concentration. Panels show micrographs of Hela cells, which were transduced with actin-RFP treated for 2 h, 4 h, 24 h and 48 h with all ten compounds at the concentrations of IC_80_ (**A**) and IC_50_ (**B**). Actin-binding agent latrunculin B induced the change of cell morphology and extensive depolymerization of the actin network. Few changes were observed in the treatment of other alkaloids. Bar = 10 μm.

To investigate potential interactions between alkaloids and actin filaments, we visualized cellular actin filaments in Hela cells using immunofluorescence staining (Figure 3 and Figure 4) and the CellLight^®^ Actin-RFP BacMam 2.0 system (Figure 6). We observed that only sanguinarine, chelerythrine and chelidonine affected actin filaments at a high concentration (IC_80_), leading to a rapid change of cell morphology and significant reduction of actin filament mass (Figure 6). No apparent changes of actin filaments were discovered in cells treated with colchicine, vinblastine, paclitaxel, noscapine, protopine or homoharringtonine.

### 2.5. Cell Cycle Analysis

**Figure 7:**
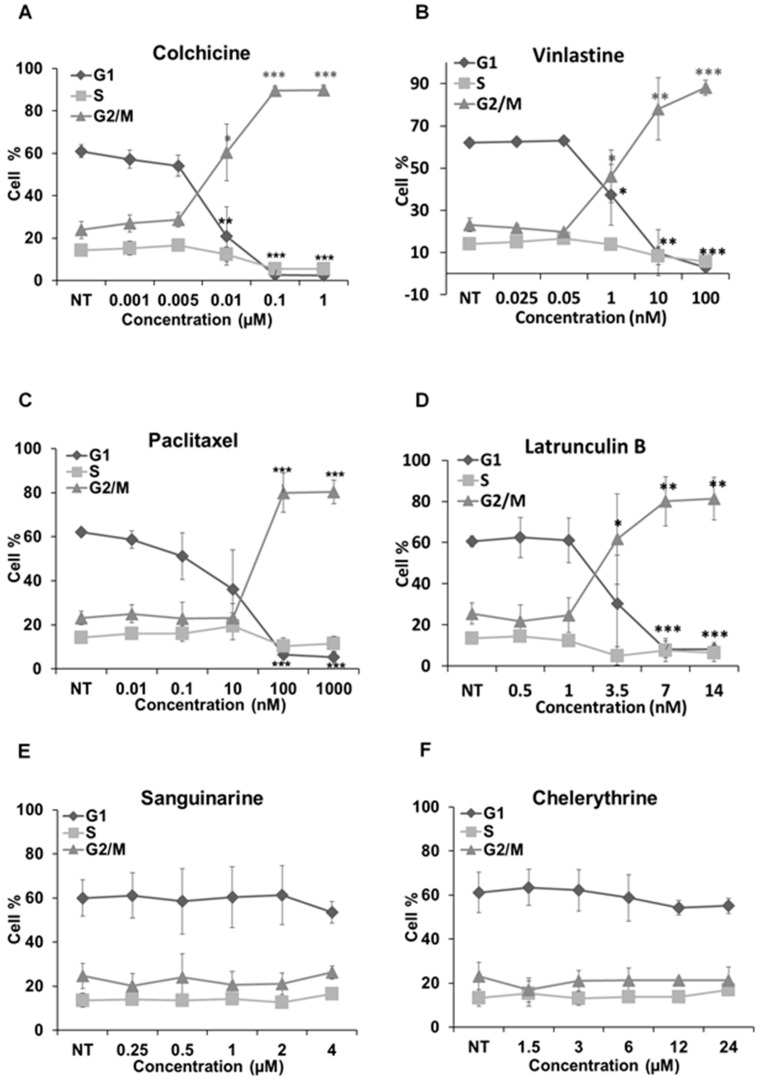

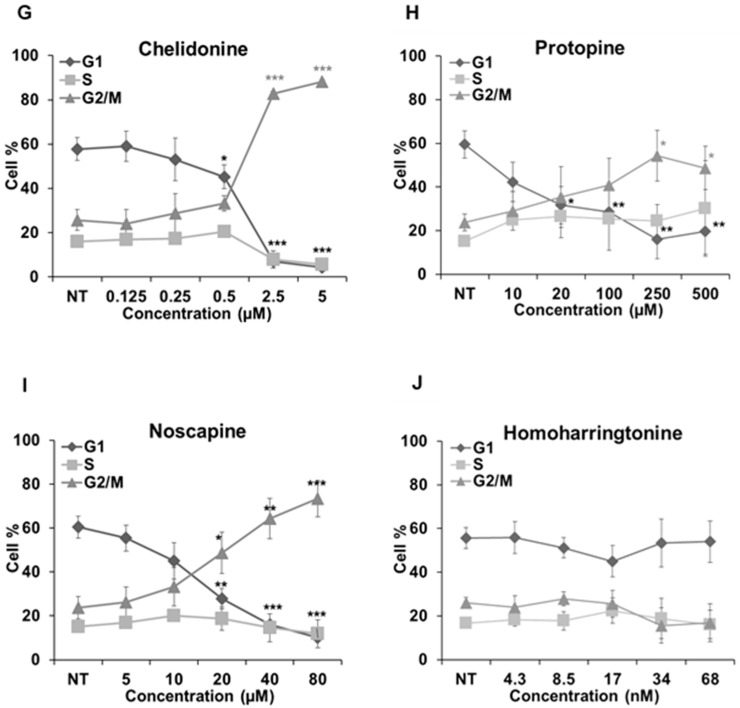
Cell cycle analysis in Hela cells. Cells were harvested after 24 h of drug treatment and subsequently assayed for their DNA content by flow cytometry. (**A**–**D**,**G**–**I**) The G2/M arrest triggered by colchicine, vinblastine, paclitaxel, latrunculin B, chelidonine, protopine and noscapine. Sanguinarine, chelerythrine and homoharringtonine did not arrest the cell cycle, which is shown in (**E**–**J**), respectively. Data are represented as the mean ± SD from three independent experiments. * *p* < 0.05, ** *p* < 0.01, *** *p* < 0.001.

We further analyzed how these alkaloids can influence the cell cycle in Hela cells (Figure 7). Known MBAs, such as colchicine, vinblastine and paclitaxel, induced a dose-dependent increase of G2/M cells with a concomitant reduction in the proportion of cells in the G1 and S phases. Quantification of the flow cytometry plots showed that 0.1 μM of colchicine significantly promoted the G2/M population from 23.91% ± 4.06% to 89.66% ± 2.04% (*p* < 0.001); 10 nM of vinblastine enhanced the G2/M population from 23.16% ± 3.15% to 78.04% ± 14.78% (*p* < 0.01); and 0.1 μM of paclitaxel from 23.12% ± 3.1% to 80.37% ± 5.36% (*p* < 0.001).

The actin-binding agent latrunculin B significantly promoted the G2/M population from 25.57% ± 5.17% to 80.05% ± 11.89% (*p* < 0.01) at the concentration of 7 μM. Sanguinarine and chelerythrine did not change the proportion of mitotic cells, though they both inhibited tubulin polymerization in vitro (Figure 5). In contrast, 2.5 μM chelidonine arrested the cell cycle in the G2/M phase with an increase from 25.67% ± 4.73% to 88.27% ± 0.99% (*p* < 0.001). Only a high concentration of noscapine (80 μM) increased the number of G2/M cells from 23.71% ± 5.03% to 73.42% ± 8.31% (*p* < 0.001), while 250 μM of protopine increased the G2/M population from 23.74% ± 3.82% to 54.35% ± 11.26% (*p* < 0.05). The cell cycle results of noscapine and protopine are in agreement with their effects on mitotic spindles (Figure 4), though they did not inhibit tubulin polymerization in vitro (Table 2). Homoharringtonine had no impact on the cell cycle, which is consistent with previous findings.

## 3. Discussion

The present study clarifies the interactions of six alkaloids and four drugs with the elements of the cytoskeleton, such as microtubules and actin filaments. Except homoharringtonine, all other alkaloids apparently affected the dynamics of microtubules, while sanguinarine, chelerythrine and chelidonine affected actin filaments in addition.

Colchicine and vinblastine are microtubule-binding agents (MBAs) that depolymerize microtubules or prevent tubulin assembly. MBAs can alter the dynamic of mitotic spindles during mitosis, which triggers the cell cycle checkpoint and thus arrests the cell cycle in the G2/M phase [10]. These can explain the effects of colchicine and vinblastine on tubulin polymerization and mitotic spindles observed in our study (Figure 4, Figure 5 and Figure 6). Latrunculin B, the actin-binding agent that destabilizes actin filaments by binding to G-actin, did not affect tubulin assembly and mitotic spindles during the study; however, it blocked the cell cycle in the G2/M phase. Cdc25 has been reported to be involved in cell size monitoring via a checkpoint mechanism during mitosis [27,28,29]. Latrunculin B can dramatically alter cell morphology (Figure 2 and Figure 6), by activating the checkpoint linked to Cdc25, and thus, blocks the cell cycle in the G2/M phase.

Paclitaxel was shown to promote the polymerization of cellular microtubules in living cells and the nucleation of tubulin assembly in vitro (Figure 2, Figure 3, Figure 4 and Figure 5), which agrees with the literature that paclitaxel stabilizes microtubules and inhibits depolymerization by binding along the polymerized microtubule [10,15]. However, we found that paclitaxel also affected the growth phase and inhibited tubulin assembly in the presence of MAPs in the tubulin polymerization assay. Paclitaxel can promote tubulin nucleation and assembly in the absence of MAPs and GTP [30,31,32]. In addition, paclitaxel does not replace the MAPs on tubulin and affect microtubule growth if MAPs firstly interact with tubulin during the polymerization [31]. Hence, we assume that a certain interaction might exist between paclitaxel and free MAPs, which would decrease the concentration of free MAPs and subsequently affect tubulin assembly.

Consistent with recent studies [33,34,35], the benzophenanthridine alkaloids sanguinarine, chelerythrine and chelidonine were found to depolymerize the microtubule network in living cells (Figure 2, Figure 3 and Figure 4) and inhibit tubulin polymerization in vitro (Table 2, Figure 5). As reported by Wolffand Knipling [33], sanguinarine, chelerythrine and chelidonine possibly bind to the colchicine site as moderate inhibitors of colchicine binding. Compared to the data of colchicine and vinblastine from our study, however, sanguinarine and chelerythrine did not cause mitotic arrest, and the way by which they affected microtubules is unique. These results agree with the same findings from other authors [35,36], which further demonstrates that sanguinarine and chelerythrine act in a different mechanism from other microtubule-binding agents, and cell cycle arrest is not their main mechanism to induce apoptosis. It should be recalled that sanguinarine and chelerythrine can intercalate DNA and via the DNA disturbance induce apoptosis [1,37]. The microtubules are composed of tubulin heterodimers that contain 20 accessible cysteine residues [38]. It is believed that the -SH groups play an important role during the polymerization, and the blocking of -SH groups can lead to the loss of polymerization competence [38,39]. Depending on the nucleophilic attack, the iminium bond C=N+ of sanguinarine and chelerythrine is susceptible to interact with tubulin by forming a reversible adduct between the iminium ion and the -SH groups [33,40]. This could be the mechanism by which sanguinarine and chelerythrine interact with tubulin and the reason why they differ from other microtubule-binding agents.

However, chelidonine cannot form a pseudobase with tubulin, like sanguinarine and chelerythrine. Chelidonine has been reported to inhibit colchicine binding to tubulin, but does not inhibit podophyllotoxin binding [33]. Based on our results, chelidonine was shown to be partially similar to colchicine in that they both depolymerized and reduced the mass of cellular microtubules and arrested cell cycle in the G2/M phase (Figure 2, Figure 3, Figure 4 and Figure 5). In addition, our data have revealed a direct relationship between cytotoxicity and cell cycle arrest in colchicine/chelidonine-treated cells (Table 1, Figure 7), and the cell cycle arrest may be due to the inhibition of tubulin polymerization. These results raise the possibility that chelidonine inhibits tubulin polymerization by binding to the domain, which is located at the colchicine site on tubulin. Generally, our findings suggest that chelidonine acts as a microtubule-destabilizing agent and that the ability of chelidonine to induce apoptosis mainly depends on its alteration on microtubules.

Besides microtubules, sanguinarine, chelerythrine and chelidonine were found to exhibit a certain effect on cellular actin filaments; in addition, the mass of actin filaments was reduced. Sanguinarine has been reported to alter protein expression of actin 87E and actin-3 [41] and induced in vitro polymerization of globular actin from rabbit muscle [42], while chelidonine has been reported to affect cell spreading and reorganization of the actin cytoskeleton in MDA-MB-231 cells [43]. These published data also indicate the potential interactions between actin filaments and benzophenanthridine alkaloids. However, it is still not clear how they act on actin filaments.

Noscapine appears to act as a microtubule-modulating agent and an antimitotic agent in this study. Noscapine significantly affected the microtubule network and spindles in living cells (Figure 2, Figure 3 and Figure 4) and arrested the cell cycle in the G2/M phase (Figure 7); however, in contrast to other MBAs, such as colchicine, it neither stabilizes nor destabilizes microtubules during polymerization in vitro. Noscapine has been reported to alter the steady state of microtubule dynamics by binding stoichiometrically to tubulin to extend the duration of the attenuated phase without changing the tubulin polymer/monomer ratio, in which the status of microtubules cannot be monitored [44,45]. This could explain the observations of our study. Although the alteration of microtubule polymerization does not occur, a slight change of the microtubule dynamics caused by noscapine is enough to activate the spindle checkpoint and induce apoptosis [10,45,46]. Due to its unique effect on microtubules, noscapine does not inhibit the functions of microtubules, such as transportation, and thus, cause low toxicity in normal cells [16,47]. Taken together, noscapine can be referred to as an anti-mitotic agent with potential antitumor application in clinical therapies.

Protopine was found to enhance the assembly of microtubules and spindles in living cells (Figure 2, Figure 3 and Figure 4) and to block the cell cycle in the G2/M phase (Figure 7). However, the way protopine acted on microtubules differs from that of paclitaxel. Chen, et al. [48] also observed the enhancement of microtubule assembly caused by protopine in human hormone refractory prostate cancer (HRPC) cells and proved this promotion through the in vitro tubulin polymerization assay. However, protopine was shown to neither promote nor inhibit tubulin assembly in vitro during our study (Table 2). How could we explain this? Compared to other alkaloids, we noticed that the mode of action of protopine is similar to that of noscapine. Protopine probably binds to tubulin without affecting its polymerization, which alters microtubule dynamics and activates the spindle checkpoint to block the cell cycle. However, further investigations are required to determine the interactions between protopine and tubulin. Therefore, we report that protopine acts as a novel anti-mitotic agent, which probably alters microtubule dynamics without affecting tubulin polymerization.

Homoharringtonine is a known protein synthesis inhibitor that has been used clinically to treat chronic myeloid leukemia [24,49]. Homoharringtonine displayed the second strongest cytotoxicity among the alkaloids during the study. However, in the following experiments, no notable alterations were observed on microtubules, cell cycle and actin filaments after the treatment of homoharringtonine, which suggests that homoharringtonine exerts its cytotoxicity without affecting the pathways related to microtubules, actin filaments and cell cycle, probably by inducing apoptosis.

## 4. Materials and Methods

### 4.1. Materials

Hela human cervical cancer cells were purchased from ATCC (Wesel, Germany), and MCF-7 human breast cancer cells were provided by Prof. Dr. Stefan Wölfl (Institute of Pharmacy and Molecular Biotechnology, Heidelberg University, Heidelberg, Germany); U2OS human osteosarcoma cancer cells, which were stably transfected with an α-tubulin-GFP construct, were supplied by Prof. Dr. Thomas Efferth (Institute of Pharmacy and Biochemistry, Johannes Gutenberg University, Mainz, Germany). Chelerythrine, chelidonine, protopine and homoharringtonine were purchased from Baoji Herbest Bio-Tech Co., Ltd. (Baoji, Shannxi, China); sanguinarine came from Chengdu Biopurify Phytochemicals Ltd. (Chengdu, Sichuan, China); G418, Atto 390 phalloidin, fetal bovine serum (FBS), 3-(4,5-dimethylthiazol-2-yl)-2,5-diphenyltetrazolium bromide (MTT), dimethyl sulfoxide (DMSO), paraformaldehyde, propidium iodide and RNase A, bovine serum albumin (BSA), piperazine-*N*,*N*′-bis(2-ethanesulfonic acid) (PIPES), EDTA, EGTA, ATP, GTP and Coomassie blue were obtained from Sigma-Aldrich Chemie GmbH (Steinheim, Germany); and mowiol^®^ 4-88 from Carl Roth GmbH & Co. KG (Karlsruhe, Germany); Dulbecco’s Modified Eagle’s Medium (DMEM), non-essential amino acids, penicillin-streptomycin, CellLight^®^ actin-RFP BacMam 2.0 actin-RFP and trypsin-EDTA came from Life technologies (Paisley, Scotland, UK) and triton X-100 from Merck KgaA (Darmstadt, Germany); mouse anti-α-tubulin monoclonal IgG and goat anti-mouse IgM-FITC were obtained from Santa Cruz Biotechnology (Heidelberg, Germany); 96-well plates, 24-well plates and 6-well plates were purchased from Greiner (Frickenhausen, Germany); and circular glass coverslips from Thermo Scientific (Braunschweig, Germany).

### 4.2. Cell Culture

Hela and MCF-cells were grown in DMEM with 10% FBS, 1% non-essential amino acids and 1% penicillin-streptomycin at 37 °C and 5% CO_2_. U2OS human osteosarcoma cancer cells were grown in DMEM with 10% FBS, 1% penicillin streptomycin and continuously treated with 250 μg/mL geneticin at 37 °C and 5% CO_2_. All experiments were performed with the cells in their logarithmic growth phase.

### 4.3. Cytotoxicity Assay

The anti-proliferative effects of alkaloids were assessed using the MTT assay, as previously described [50]. In brief, 1 × 10^4^ cells were seeded in 96-well-plates and incubated with serial dilutions of alkaloids for 48 h (Hela, U2OS) and 72 h (MCF-7). MTT solution was then added and incubated for 2 h. After the addition of DMSO, the plate was read at 570 nm using the Tecan infinite M200 Pro (Tecan, Crailsheim, Germany).

### 4.4. Imaging of Tubulin-GFP Transfected U2OS Cells

α-Tubulin-GFP U2OS cells (1 × 10^5^) were seeded in 24-well plates and grown for 24 h. Then, 200 μL different concentrations of alkaloids (IC_80_, IC_50_) were added into each well, and the cells were imaged under a Keyence BZ-9000 microscope (Keyence, Neu-Isenburg, Germany) after incubation for 2 h, 4 h, 24 h and 48 h. The images were analyzed using BZ-II Analyzer software (version 2.1, Keyence, Neu-Isenburg, Germany).

### 4.5. Immunofluorescence Staining

Hela cells (1 × 10^5^) were seeded on sterile circular glass coverslips and grown in the absence and presence of different concentrations of alkaloids (IC_80_, IC_50_) for 24 h. Then, cells were fixed in 4% paraformaldehyde at 4 °C for 20 min and permeabilized by 0.3% (*v*/*v*) Triton X-100 for 10 min. Nonspecific binding sites on the coverslips were blocked by incubating them with 5% FBS for 1 h. To stain the microtubules, cells were firstly incubated with 1:100 mouse anti-α-tubulin monoclonal IgG for 1 h and then with 1:200 goat anti-mouse IgM-FITC for 1 h. Cells were then incubated with 20 nm/mL Atto 390 phalloidin for 45 min to stain the actin filaments and 20 μg/mL propidium iodide for 30 min to stain the cell nucleus/chromosomes. After washing steps, the coverslips were mounted by mowiol. Microtubules, actin filaments and cell nucleus/chromosomes were observed and analyzed using the Keyence BZ-9000 microscope as described before.

### 4.6. Imaging of Actin-RFP Transfected Hela Cells

Hela cells (2 × 10^4^) were seeded in 24-well plates and mixed with CellLight^®^ Actin-RFP BacMam 2.0, which is a fusion construct of human actin and TagRFP, providing an accurate and specific targeting to cellular actin filaments. After 16 h of incubation, 200 μL of different concentrations of alkaloids (IC_80_, IC_50_) were added into each well, and the cells were analyzed as described above (Section 4.4).

### 4.7. In-Vitro Tubulin Polymerization Assay

Tubulin plus microtubule-associated proteins (MAPs) were prepared from porcine brains (freshly obtained from a local slaughter house) by two cycles of polymerization and depolymerization according to a standard protocol [51]. Tubulin polymerization assays were carried out in polymerization buffer (100 mM PIPES, 2 mM EGTA, 0.1 mM EDTA, 3 mM MgCl_2_, 1 mM ATP and 1 mM GTP, pH 6.85) by mixing 5.6 mg/mL tubulin-MAPs with different concentrations of alkaloids and incubating at 37 °C for 40 min. The rate and extent of the polymerization reaction were monitored by light scattering at 360 nm using Tecan infinite M200 Pro.

### 4.8. Cell Cycle Analysis

Cell cycle analysis was carried out as established in our laboratory [52]. Hela cells (5 × 10^5^) were seeded in 6-well plates and treated with different concentrations of alkaloids for 24 h. Cells were then collected, centrifuged and fixed in 70% ice-cold ethanol for at least 8 h. After the washing steps, cells were treated with 0.2 mg/mL RNase A for 30 min at 37 °C and then stained with 0.1 mg/mL propidium iodide. Samples were analyzed using a FACScan flow cytometer (Becton Dickinson, Heidelberg, Germany). Data were analyzed using Cell Quest™ Pro software (Becton Dickinson) and Microsoft Excel 2013 (Microsoft Corporation, Redmond, WA, USA).

### 4.9. Statistical Analysis

The IC_50_ was determined as the amount of the substances needed to reduce 50% cell viability/tubulin polymerization and calculated from concentration-response curves by Sigmaplot software (Systat Software Inc., San Jose, CA, USA). All experiments were performed at least three times. Data are presented as the mean ± standard deviation. Statistical comparison between controls and different treatments were performed by an unpaired Student’s *t*-test. Significance was considered at *p* < 0.05.

## 5. Conclusions 

This study systematically investigated the roles of these cytotoxic alkaloids in biological processes related to cytoskeletal proteins, especially in mitosis, tubulin polymerization and cell cycle. In conclusion, we found that: (1) paclitaxel can inhibit microtubule disassembly and tubulin polymerization in vitro; (2) sanguinarine and chelerythrine are anti-tubulin-assembly agents, which do not arrest the cell cycle; (3) chelidonine is a microtubule-destabilizing agent, which acts in a different mode than sanguinarine and chelerythrine; (4) protopine and noscapine are novel anti-mitotic agents without affecting tubulin assembly in vitro. Our findings challenge the classic roles of these alkaloids and suggest that cytoskeletal interference could be partly responsible for their cytotoxic properties. These results may also allow a more comprehensive understanding of the changes of cancer cells responding to these alkaloids. More studies at a molecular level are necessary to better understand these results.

## Figures and Tables

**Table 1:** Cytotoxic activity of alkaloids and reference drugs *, ** against Hela, MCF-7 and U2OS cancer cells.

Compounds	IC_50_		
	Hela	MCF-7	U2OS
Colchicine *	0.01 ± 0.01 μM	0.02 ± 0.01 μM	0.01 ± 0.01 μM
Vinblastine *	0.02 ± 0.01 nM	0.07 ± 0.05 nM	0.10 ± 0.05 nM
Paclitaxel *	0.08 ± 0.10 nM	1.05 ± 0.43 μM	0.23 ± 0.10 μM
Latrunculin B **	7.13 ± 0.61 μM	36.78 ± 2.25 μM	5.48 ± 0.68 μM
**Sanguinarine**	1.03 ± 0.04 μM	1.24 ± 0.21 μM	0.92 ± 0.53 μM
**Chelerythrine**	3.85 ± 2.10 μM	7.63 ± 3.43 μM	2.88 ± 0.76 μM
**Chelidonine**	1.13 ± 0.37 μM	3.85 ± 0.10 μM	3.86 ± 1.99 μM
**Protopine**	27.50 ± 11.77 μM	67.28 ± 9.06 μM	62.36 ± 9.90 μM
**Noscapine**	16.81 ± 9.79 μM	35.20 ± 8.18 μM	60.85 ± 12.17 μM
**Homoharringtonine**	9.03 ± 0.38 nM	0.87 ± 0.45 nM	3.0 ± 1.67 nM

* Active on tubulin/microtubules; ** active against actin filaments; data are presented as the mean ± the standard deviation (SD).

**Table 2:** Inhibition of tubulin polymerization in vitro.

Compounds	IC_50_
**Colchicine**	2.89 ± 0.36 µM
**Vinblastine**	1.42 ± 0.05 µM
**Paclitaxel**	38.19 ± 3.33 μM
**Latrunculin B**	>1 mM
**Sanguinarine**	48.41 ± 3.73 μM
**Chelerythrine**	206.39 ± 4.20 μM
**Chelidonine**	34.51 ± 9.47 μM
**Protopine**	>1 mM
**Noscapine**	>1 mM
**Homoharringtonine**	>1 mM

Data are presented as the mean ± the standard deviation (SD).
